# Expression of caveolin in trabecular meshwork cells and its possible implication in pathogenesis of primary open angle glaucoma

**Published:** 2011-11-09

**Authors:** Irina Surgucheva, Andrei Surguchov

**Affiliations:** 1 Laboratory of Retinal Biology, Veterans Administration Medical Center, Kansas City, MO; 2 Department of Neurology, Kansas University Medical Center, Kansas City, KS

## Abstract

**Purpose:**

Primary open-angle glaucoma (POAG), which is the most common form of glaucoma, has been associated with a heterogeneous genetic component. A genome-wide association study has identified a common sequence variant at 7q31 (rs4236601 [A]) near the caveolin genes in patients with POAG. Caveolins are a family of integral membrane proteins which participate in many cellular processes, including vesicular transport, cholesterol homeostasis, signal transduction, cell adhesion and migration. The goal of this study was to investigate the expression and regulation of caveolin 1 (*CAV-1*) and caveolin 2 (*CAV-2*) in normal and glaucoma trabecular meshwork (TM) cells.

**Methods:**

CAV-1 and CAV-2 protein expression was quantified by immunoblot analysis using lysates isolated from primary and immortalized TM cells or TM tissue dissected from normal and POAG eyes. The localization of caveolins in TM cells was assessed by immunofluorescent microscopy. CAV-1 and CAV-2 protein expression was also investigated in TM cells at various time points after subjecting the cells to known glaucomatous insults like dexamethasone (DEX) and tumor growth factor beta2 (TGF-β2) treatment. Phosphorylation of CAV-1 at tyrosine 14 in normal and glaucoma TM cell lines was evaluated using a specific monoclonal antibody (Ab). The 5′ upstream region of the *CAV-1* gene was amplified and the sequence variant rs4236601 (A/G polymorphic site) and several putative transcription factor-binding sites were modified by in vitro mutagenesis. The effect of nucleotide sequence modifications in the *CAV-1* upstream region on gene expression was assayed in a luciferase-based system in TM and non-TM cells.

**Results:**

*CAV-1* and *CAV-2* are expressed in TM cells, with localization to the cytoplasm and perinuclear region. DEX increased CAV-1 expression in immortalized glaucoma TM cells by 2.8±0.1 (n=3) fold at 24 h and 2.5±0.1 (n=3) fold at 48 h, compared to 1.3±0.06 (n=3) fold at 24 and 48 h in immortalized normal TM cells. Phosphorylation of CAV-1 at Tyr14 was reduced by 3.2±0.15 (n=3) fold in glaucomatous TM cells when compared to normal TM cells. In POAG and normal TM tissue, CAV-1 expression was found to be uniform. CAV-2, on the other hand, was variable in independent normal and glaucoma TM tissue. Substitution of a G for an A at base pair −2,388 upstream of the start codon of *CAV-1*, corresponding to the minor allele rs4236601 [A], increased transcriptional activity in TM and non-TM cells when compared to the native sequence. Deletion analysis of putative transcription factor binding sites in the *CAV-1* promoter region caused cell-specific effects on gene expression.

**Conclusions:**

*CAV-1* and *CAV-2* are expressed in normal and glaucoma tissue and TM cell lines. Phosphorylation of Tyr14 in CAV-1 and transcriptional regulation of *CAV-1* expression may have a role in glaucomatous alterations in TM cells.

## Introduction

Primary open angle glaucoma (POAG), which is the most common form of glaucoma, is the second leading cause of blindness worldwide, affecting nearly 70 million people [1]. POAG usually affects older people and, with the aging of world populations, it is predicted that the number of patients with the disease will substantially increase over the next decade [2]. The prevalence of POAG among Americans aged 40 and older is estimated at 1.86 percent, affecting 2.22 million individuals [3].

The disease mainly affects the optic nerve and retinal ganglion cells leading to visual impairment and blindness. Of the various risk factors of POAG, elevated intraocular pressure (IOP) is the most prevalent and clinically relevant. The elevation of IOP results from impaired drainage of aqueous humor through the conventional pathway, which consists of the trabecular meshwork (TM), Schlemm’s canal (SC), collector channels and aqueous veins [3,4].

Recently, a genome-wide association study identified the first common genetic risk factor for POAG, corresponding to a region of the genome on chromosome 7q31 [5]. The locus spans the caveolin 1 (*CAV-1*) and caveolin 2 (*CAV-2*) genes and the minor allele of a genetic marker, rs4236601, located in the noncoding upstream region of *CAV-1*, was found to be tightly associated with POAG. However, a smaller population based study suggests that the rs4236601 polymorphic allele may not be a universal risk factor in all populations [6]. Although the association does not mean that caveolins are directly implicated in glaucoma, the finding has prompted interest in caveolin family members and their role in cells of the conventional outflow pathway.

Caveolins are the signature proteins of caveolae, flask-shaped invaginations of plasma membrane that are 50–100 nanometers in diameter (reviewed in [7-9]). Caveolins are scaffolding proteins with molecular weight 22–24 kDa embedded in the cytosolic leaflet of cell membranes, with both NH_2_- and COOH-termini residing in the cytosol [10,11]. Caveolins interact with and compartmentalize membrane-localized signaling proteins to facilitate and modulate high-fidelity intracellular signaling pathways. In addition, they participate in many important cellular processes such as vesicular transport (including cell to cell protein and microRNA delivery), adhesion and cell motility, cholesterol homeostasis and tumor suppression [7-9]. Caveolin structure, function and role in human diseases have been extensively investigated in different tissues and organs [9-13].

**Figure 1:**
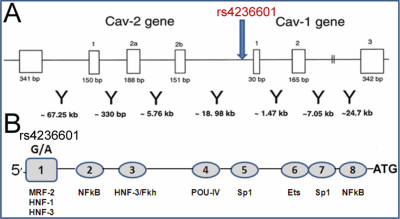
Organization of the human CAV-1 and CAV-2 genes and localization of TF binding sites. **A**: Exon-intron structure of the caveolins genes and localization of SNP rs4236601 marker (modified from [19]). The sizes of exons (white rectangular boxes) and the distance between them (below Y) are shown. Marker rs4236601 is located 2,388 bp upstream from the start codon of *CAV-1*. **B**: localization of SNP and putative TF binding sites (ovals) in the upstream region of *CAV-1*. Other details of TF-binding sites 1–8 are present in Table 2.The sequence of primers used for the modifications of the fragment are presented in Table 1.

There are three caveolin proteins: CAV-1, CAV-2, and caveolin 3 (CAV-3) [12-15] which form a structural backbone of caveolae. CAV-1 and CAV-2 have overlapping patterns of expression throughout numerous tissues [16,17], whereas CAV3 has more restricted expression in muscle [15,18] and in the nervous system [17]. *CAV-1* and *CAV-2* genes are located within 19 kilobases on human chromosome 7q31.1 (Figure 1), while *CAV-3* is mapped to chromosome 3p25 [19]. Several cell-type specific transcription factor (TF) binding sites ensuring cell and tissue-specific expression have been described in the caveolin promoter region [20-24]. Due to the reported genetic association of a marker near *CAV-1* and *CAV-2* with POAG, we analyzed their expression and regulation in normal and glaucomatous TM cells. Additionally, we evaluated the role of cis-elements upstream region of *CAV-1* and the effect of the recently described G-A polymorphism located in the upstream region of *CAV-1* (Figure 1) in transcriptional regulation.

## Methods

### Normal and POAG TM tissue

TM tissues were dissected from normal and POAG donor eyes. Tissues were placed in lysis buffer (50 mM Tris pH 8.0, 0.5% sodium dodecyl sulfate, 0.5% Triton-X100, 137 mM NaCl, 3 mM KCl, 8 mM Na_2_HPO_4_-7H_2_O, 1 mM KH_2_PO_4_, protease inhibitors [Roche, Indianapolis, IN]) [25] and homogenized using a Polytron rotor stator homogenizer (Kinematica, Lucerne, Switzerland). Supernatant was cleared of cellular debris by centrifugation at 10,000× g for 5 min. Total protein concentration of cleared lysates was estimated using a Bradford assay (Bio-Rad, Hercules, CA). Remaining lysate was stored at −80 °C until further use.

### Human TM cell culture

SV-40 transformed human normal (NTM-5) and glaucoma (GTM-3) TM cell lines were derived from normal and glaucoma donor eyes, respectively (generous gifts from Dr. Abbott F. Clark, Alcon Laboratories, Fort Worth, TX) [26,27]. The cells were cultured to confluence in Dulbecco’s Modified Eagle’s Medium (DMEM, Cat. # D5671; Sigma, St. Louis, MO) containing 4 mM glutamine (Gibco, Carlsbad, CA), 10% fetal bovine serum (FBS; Equitech Bio, Inc., Kerrville, TX), 100 U penicillin and 100 μg/ml streptomycin (Cellgro, Manassas, VA). NTM-5 and GTM-3 cell cultures were maintained at 37 °C under a humidified atmosphere of 5% CO_2_/95% air.

Primary cultures of TM cells (n=2) were established from two separate donor eyes (a 20-year-old female and a 32-year-old female) as described previously [28]. Briefly, TM was dissected and digested in collagenase A (Worthington Biochemical Corporation, Lakewood, NJ). The cells were pelleted, resuspended in DMEM (Mediatech, Inc., Herndon, VA), supplemented with 10% FBS and 100 U penicillin and 100 μg/ml streptomycin and placed in 6 well plates until the cells were confluent. Cells from passages 3 to 7 were used for the experiments.

### Treatment of TM cells with DEX and TGF-β2

Confluent NTM cells were serum-starved overnight and treated with either 100 nM dexamethasone (DEX; Sigma) or transforming growth factor beta 2 (TGF-β2; Sigma). Following treatment, cells were collected at various time points and lysates prepared as described previously [29]. Briefly, for DEX treatment, 10^-7^ M DEX was added to TM culture grown in serum free media. Cells and CM were collected at 24 and 48 h and proteins were separated on 12% SDS-PAGE gels. TGF-β2 was used at concentration 5 ng/ml.

### Immunoblot analysis

Monoclonal mouse anti-CAV-1 (Cat. #610406), polyclonal rabbit Anti-CAV-1 (Cat. #610059), monoclonal mouse anti-phospho Tyr14-CAV-1 (pY14, Cat. # 611338), and monoclonal mouse anti-CAV-2 (Cat. # 610684; GE Healthcare Bio-Sciences Corp., Piscataway, NJ) were purchased from BD Transduction Laboratories (San Jose, CA). Actin monoclonal mouse Ab (Cat# MAB1501; GE Healthcare Bio-Sciences Corp.) was obtained from Chemicon International (Temecula, CA). ECL anti-mouse IgG, horseradish peroxidase-linked species-specific whole Ab (Cat. #NA931) or ECL anti-rabbit IgG, horseradish peroxidase-linked (Cat# RPN2108) were used as secondary Abs for western blot. Goat anti-rabbit IgG-conjugated by AlexaFlor 488 (Invitrogen, Carlsbad, CA) was used for the detection of CAV-1. Goat anti-mouse IgG Rhodamine Red-X (Jackson ImmunoResearch Lab., West Grove, PA) conjugated was used for CAV-2 detection.

Protein fractions (10 μg) from lysed cells or tissues were resolved in 12% SDS-polyacrylamide gels and transferred onto PVDF membranes. Blots were incubated in mouse anti-Cav-1 and anti-Cav-2 primary Abs followed by the incubation in HRP-conjugated secondary antibody. Enhanced chemiluminescent substrates (ECL Plus, GE Healthcare, Piscataway, NJ) were used to visualize the bands. Differential changes in caveolin expression were calculated by densitometric analysis using Kodak Molecular Imaging software, (Woodbridge, CT) Version 4.5. Other details of immunoblotting are described previously [30,31]. Briefly, 15-20 μg of total protein extract were loaded on a 12% polyacrylamide gel. After electrophoresis, proteins were transferred onto 0.45 μm polyvinylidene fluoride (PVDF) membrane (Millipore, Billerica, MA). Nonspecific binding sites were blocked by immersing the membrane in a 5% blocking reagent in Tris-buffered saline Tween (TBS-T) for 1 h. Membranes were washed, incubated with antibody, and exposed to the film as described by the manufacturers of ECL western blotting detection reagents (Amersham Pharmacia Biotech, Piscataway, NJ). Fold change is represented by the means±SEM from four independent experiments.

### Immunofluorescent staining

NTM-5 and GTM-3 cells were grown on coverslips coated with Poly-D-lysine. The cells were washed with cold PBS and fixed in 4% paraformaldehyde for 15 min at room temperature. Subsequently, the cells were washed and incubated in blocking buffer (3% BSA, 3% NGS, 0.1% triton X-100 in PBS) for 2 h at room temperature. The coverslips were put in respective primary Ab solutions overnight at 4 °C. Following incubation, coverslips were washed with cold PBS and placed in the appropriate secondary Ab for 1 h at room temperature. Coverslips were washed with cold PBS and mounted on slides with Vectashield containing DAPI (Vector Laboratories, Burlingame, CA) [32,33]. Images were digitally captured using an Olympus 1X71 inverted microscope (Olympus, Waltham, MA), with 40× magnification.

### Immunoprecipitation (IP)

For CAV-1 IP, 4 μl of polyclonal rabbit anti-CAV-1 with concentration 250 μg/ml from BD Transduction Laboratories (San Jose, CA) was used. IP was performed from cell extract containing 300 μg of total protein using Protein A agarose beads as described in the ABCam Immunoprecipitation Protocol. After IP, the samples were dissolved in 2× loading buffer, separated on SDS-12% polyacrylamide gel, transferred to PVDF membrane and probed with monoclonal mouse anti-CAV-1(Cat. #610406) or anti-phospho Tyrosine14-CAV-1 antibodies (Cat. # 611338) both from BD Transduction Laboratories (San Jose, CA).

### Generation of the *CAV-1* upstream region

BAC clone RP11–39K12 (Empire Genomics, Cedarhurst, NY) containing the 5′- upstream region of human *CAV-1* was used as a template for PCR [GC-rich PCR system (Roche, Indianapolis, IN)] to generate a 2,465 bp upstream region of *CAV-1* (forward primer 1 contained the KpnI site, reverse primer 2 contained the SacI site, Table 1). The 2,465 bp fragment was purified using Zymo kit (Zymo Research Corporation, Irvine, CA), digested with KpnI and SacI and inserted into the pGL3 basic vector (Promega, Madison, WI). Sequence analysis of the fragment revealed an A at position −2,388 upstream from the *CAV-1* start codon, representing the minor allele associated with POAG [5]. This construct was named pGL3-CAV-1promoterA.

**Table 1:** Primers used for the amplification and in vitro mutagenesis.

1	F 5′-TATAGGTACCAATTTATGGCAGTCATTA-3′	Amplification
2	R 5′-ATATGAGCTCGCTGGCCCGTGGCTGGAT-3′	
3	S 5′-GTCCTTTTCTAGTGTATTGTGTTTGTTAATATTTG-3′	A→G
4	Compl 5′-CAAATATTAACAAACACAATACACTAGAAAAGGAC-3′	
5	S 5′-GCTCCCACTAATGTGGTATTTAACTTTTGAAAGATCTTTAG-3′	NFκB (−1537)
6	Compl 5′-CTAAAGATCTTTCAAAAGTTAAATACCACATTAGTGGGAGC-3′	
7	S 5′-GCTTTAAATAATTCTACAATATTTTGTGTATTTTGC-3′	HNF-3
8	Compl 5′-GCAAAATACACAAAATATTGTAGAATTATTTAAAGC-3′	
9	S 5′-CAGAAAATATCGGTAATAAAAAAAGTTAAAGATCTTC-3′	Pou-IV
10	Compl 5′-GAAGATCTTTAACTTTTTTTATTACCGATATTTTCTG-3′	
11	S 5′-GGAGGCTCCCTCCCCCCCCTCCAGCGC-3′	Sp1 (−236)
12	Compl 5′-GCGCTGGAGGGGAGGGAGGGAGCCTCC-3′	
13	S 5′-GACCCGGCGCAGCACACCAACCGCGAGCAGAAC-3′	Ets
14	Compl - 5′-GTTCTGCTCGCGGTTGGTGTGCTGCGCCGGGTC-3′	
15	S 5′-CGCTGCGGGCGCTTGCTGCCAGAACCTTG-3′	Sp1 (−124)
16	Compl 5′-CAAGGTTCTGGCAGCAAGCGCCCGCAGCG-3′	
17	S 5′-CTTAAAGCACAGCCCATCACAGTTTTCATCCAGC-3′	NFκB (−40)
18	Compl 5′-GCTGGATGAAAACTGTGATGGGCTGTGCTTTAA-3′	

1 and 2 - human CAV-1 promoter-specific primers used to amplify 2, 465 bp PCR product: forward primer containing Kpn1 site and reversed primer containing SacI site. Three and 4 - primers used for the replacement of A on G in position −2,388 upstream from the start codon in CAV-1 promoter region (Figure 1B, modification 1). Primers 5 – 18 used for the deletion of putative TF binding sites. Other details are presented in Methods and Table 2.

### In vitro mutagenesis of the *CAV-1* upstream region

To generate the G variant at position −2,388, in vitro mutagenesis using the Stratagene “Quick Change Site-Directed Mutagenesis Kit” (Cat. 200518; Santa Clara, CA) was performed. Primers 3 and 4 (Table 1) were used to replace A with G at position −2,388 upstream from the start codon in the CAV-1 promoter region (Figure 1B; modification 1 in Table 2). Upstream region containing a G at position −2,388 was inserted into pGL33-basic and the resulting construct was called pGL3-CAV-1promoterG. The effect of the A-G replacement, as well as the deletions of putative TF-binding sites shown in Figure 1B (ovals) and in Table 2 on the efficiency of gene expression was evaluated in TM cells from glaucoma patient and control individuals. Since A in position −2,388 from the start codon ATG in *CAV-1* (GenBank NG_012051.1; GI: 237820664) is a minor allele associated with POAG, the results are presented in such a way that the activity of the construct with G (pGL3-CAV-1promoterG) is assigned 100% and the efficiency of the construct with A in this position (pGL3-CAV-1promoterA) is used for comparison.

**Table 2:** Modified sequences and localization of putative TF-binding sites in the 2,465 kilobase fragment used in LUC system.

**Modifications (substitutions or deletions)**	**Location in the 2,465 base pairs fragment**	**Names of putative TF-binding site**	**Reference**
1. G/A polymorphism (rs4236601)	−2,388	MRF2, HNF1, HNF3	[5]
2. gggaaaatcc	−1,537, −1,527	NFkB	[21]
3. tataaac	−889, −882	HNF-3/Fkh homolog1	[60]
4. attatag	−807, −800	POU-IV	[60]
5. ccccgccctc	−236, −226	Sp1	[50]
6. cgtccggg	−182, −174	Ets	[24]
7. cagccaccg	−124, −115	Sp1	[21]
8. gggaaacctcct	−40, −28	NFkB	[61]

### Deletions of putative TF binding sites from the *CAV-1* upstream region

For deletions of putative TF binding sites from the *CAV-1* upstream region. a Stratagene “Quick Change Site-Directed Mutagenesis Kit” (Cat. 200518; Santa Clara, CA) was used following the manufacturer’s recommended protocol. Table 1 shows the primer sequences used for the deletions of putative TF binding sites (primers 5- 18). Other details of the procedure have been described previously [34]. Briefly, the oligonucleotide primers, each complementary to opposite strands of the vector, were extended during temperature cycling by Pfu Turbo DNA polymerase. The cycling was performed as follows: 95 °C, 2 min, followed by 18 cycles at 95 °C, 20 min, 60 °C , 10 min, 68 °C 4 min 5 s. Finally the samples were incubated at 68 °C  for 4 min. The generated product was treated 15 min at 37 °C with Dpn I endonuclease to digest the parental DNA template and to select mutation-containing synthesized DNA.

### Transient transfection and LUC assays

NTM-5 and GTM-3 cells were split into 24 well plates the day before transfection at a density of 2×10^5^ cells in 500 μl of DMEM with 10% FBS without antibiotics. Transfection was performed using Lipofectamine 2000 (Invitrogen, Carlsbad, CA). Briefly, pGL3-CAV-1promoter was mixed with Lipofectamine at a ratio of 1:2.5. pRL-SV40 containing the Renilla-Luc gene (Promega, Madison, WI) was co-transfected to provide an internal standard for transfection efficiency. After a 4–6 h incubation, media containing lipofectamine and DNA was removed and replaced with DMEM with 10% FBS. The cells were incubated at 37 °C for 24 h, harvested and assayed for firefly-Luc and Renilla-Luc activities using the DRL Luc assay system (Promega, Madison, WI) as per the manufacturer’s protocol. Relative light units (RLU) were determined using 100 µl of the appropriate substrate in a BioTek microplate reader (Synergy H1; Winooski, VT) Following normalization with Renilla-LUC activity, activity of pGL3-CAV-1 promoterG was assigned 100% and the efficiency of the other constructs were used as comparisons. The LUC values are expressed as percent change with each vector. Each experiment was performed at least 3 times and the results are represented as mean±SEM. The results were analyzed by ANOVA (ANOVA).

## Results

### CAV-1 and CAV-2 expression in TM cells from POAG patients and controls

**Figure 2:**
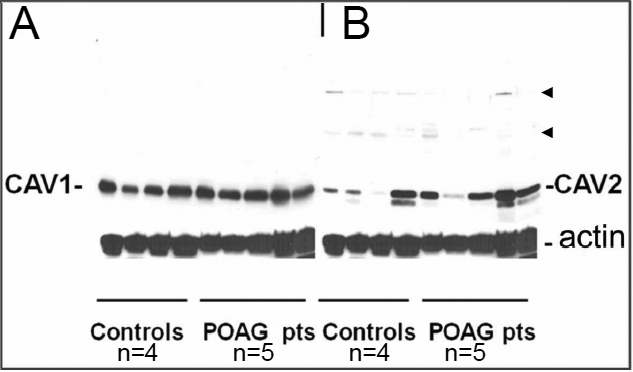
CAV-1 and CAV-2 in TM of POAG patients and controls. Total protein (20 μg) from TM cells were resolved in 12% SDS-polyacrylamide gels, transferred on PVDF membrane and probed with Abs to CAV-1 (**A**) and CAV-2 (**B**). The amount of CAV-1 was similar both in POAG patients and controls, whereas the CAV-2 was highly variable among samples. Heterooligomers CAV-1 – CAV-2 are shown by arrowheads.

TM cells from POAG patients and control individuals were used to compare the level of CAV-1 and CAV-2. According to western blot analysis, CAV-1 is present in TM from POAG patients and control samples in almost equal amounts (Figure 2A), whereas for CAV-2 considerable quantitative variability between individuals were found (Figure 2B). In addition higher molecular weight bands of CAV-2 were found which have previously been identified as hetero-oligomers containing CAV-1 and CAV-2 [35].

### CAV-1 and CAV-2 expression and localization in TM cell cultures

**Figure 3:**
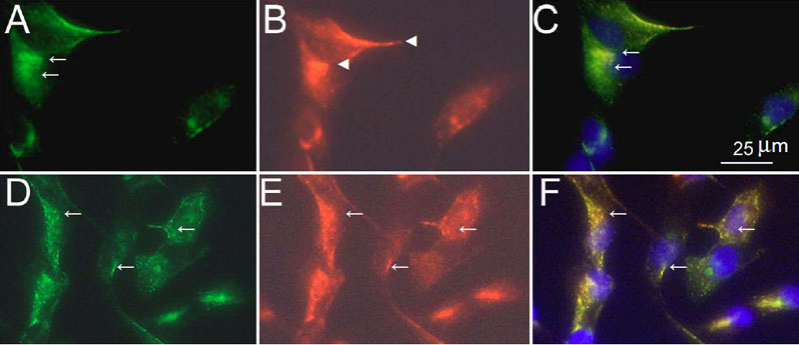
Immunofluorescence localization of CAV-1 and CAV-2 in TM cells. CAV-1 (**A, D**, green). CAV-2 (**B, E,** red). NTM cells (**A-C**). GTM cells (**D-F**). Polyclonal rabbit anti-CAV-1 and monoclonal mouse anti-CAV-2 were used for immunostaining. **C** and **F**: merged; blue – DAPI staining. CAV-1 and CAV-2 immunoreactivity is identified as punctate staining with a predominant cytoplasmic localization (arrows). In NTM cells CAV-1 was mainly found in the cytoplasm (**A,** arrows), while CAV-2 was present in cytoplasm, the perinuclear area as well as in the cell membranes (**B**, arrowheads). In GTM cells CAV-1 and CAV-2 are colocalized in dot-like structures (arrows, **D-F**).

CAV-1 and CAV-2 were found to have similar intracellular localization in normal (Figure 3A-C) and glaucomatous (Figure 3D-F) TM cells showing a perinuclear punctuate appearance. CAV-1 expression was observed in the cytoplasmic compartment (Figure 3, arrow), while CAV-2 appeared to be bound to the cell membrane (Figure 3B, arrowheads). In both cell types, there was extensive colocalization of CAV-1 and CAV-2.

### Effect of DEX on caveolin expression in TM cells

**Figure 4:**
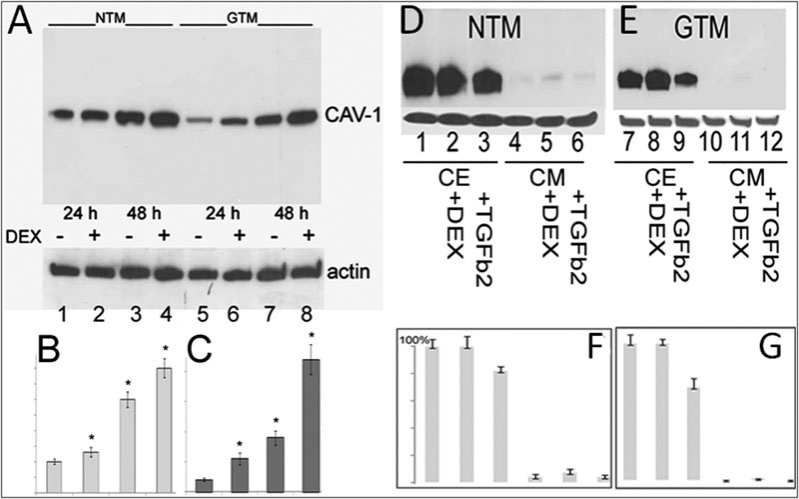
CAV-1 expression and secretion from NTM and GTM cells. **A**: DEX differentially induces CAV-1 expression in NTM-5 and GTM-3 cells after 24 and 48 h. Western blot probed with CAV-1 Ab. Below – the same blot was reprobed for actin. **B**: The first four lanes (1–4) for NTM samples shown in **A** were scanned using KODAK MI Software. **C**: The next four lanes (5–8) for GTM samples were scanned. The values indicated represent the means±SEM from four independent experiments. **D** and **E**: CAV-1 in cell extracts (CE; lanes 1–3 and 7–9) and conditioned media (CM; lanes 4–6 and 10–12) of NTM-5 and GTM-3 cells. Lanes 1 and 7 – control cells; lanes 2 and 8 – cells were incubated with 100 μM of DEX for 96 h; lanes 3 and 9 – cells incubated with 5 ng/ml of TGFβ2 for 48 h. TGFβ2 reduced CAV-1 expression to 85% in NTM-5 cells (lane 3) and to 68% in GTM-3 cells (lane 9), whereas DEX did not affect CAV-1 expression after 96 h. Low level of CAV-1 secretion was observed from NTM cells (**D**, lanes 4–6) which was increased by DEX (lane 5). No expression was observed from GTM cells (**E**, lanes10–12). **F**: Bands for NTM samples shown in **D** were scanned. **G**: Bands for GTM samples shown in **E** were scanned. The values indicated represent the means±SEM from four independent experiments.

To analyze CAV-1 expression in the presence of an agent that causes glaucomatous insults by activating mechanisms relevant to glaucoma pathogenicity, we treated immortalized NTM and GTM cells with DEX [36,37]. In GTM-3 cells incubated with 100 nM DEX the level of CAV-1 expression increased 2.75 and 2.45 folds after 24 h and 48 h of incubation, respectively, as compared to control (Figure 4A, lanes 5–8 and Figure 4C). At the same time, the effect of DEX on the level of CAV-1 expression in NTM-5 cells was significantly less prominent (1.3 fold increase after 24 h and 48 h of incubation (Figure 4A, lanes 1–4 and Figure 4B).

### Tyrosine 14 phosphorylation in CAV-1

**Figure 5:**
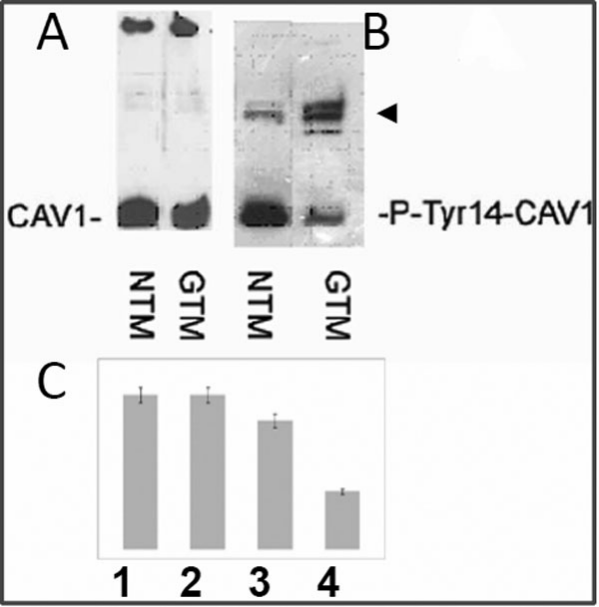
Phosphorylation of Tyr14 in CAV-1 in NTM-5 and GTM-3 cells. **A**: NTM and GTM cell lysates were immunoprecipitated by polyclonal antirabbit Ab against CAV-1 and the precipitate was suspended in a 2× loading buffer (ABCam Immunoprecipitation Protocol) and western blotted with monoclonal antimouse CAV-1 Ab. **B**: The same blot was stripped and reprobed with monoclonal antimouse pTyr14-CAV-1 antibody. **C**: Lanes shown in **A** and **B** were scanned using KODAK MI Software. The amount of non-phosphorylated CAV-1 is similar in NTM-5 and GTM-3 samples (**A**), while the amount of CAV-1 phosphorylated at Tyr14 (P-Tyr14-CAV-1) is 2.3 times lower in GTM-3 cells. Arrowhead indicates heavy chain immunoglobulins. The values indicated represent mean±SEM from four independent experiments.

The localization and functional properties of CAV-1 are modulated by phosphorylation of tyrosine-14 (Tyr14), which may alter its trafficking and accumulation at focal adhesion [38-40]. In GTM-3 cells, the level of Tyr14 phosphorylation (Figure 5B, lane 4) was 3.2 times lower than in NTM-5 cells (Figure 5B, lane 3), while the amount of non-phosphorylated CAV-1 was equal (Figure 5A, lanes 1 and 2).

### Effect of G/A polymorphism on expression

The effect of transcription of the G/A variation at −2,388 upstream of the CAV-1 transcription initiation site was examined in the LUC system.

**Figure 6:**
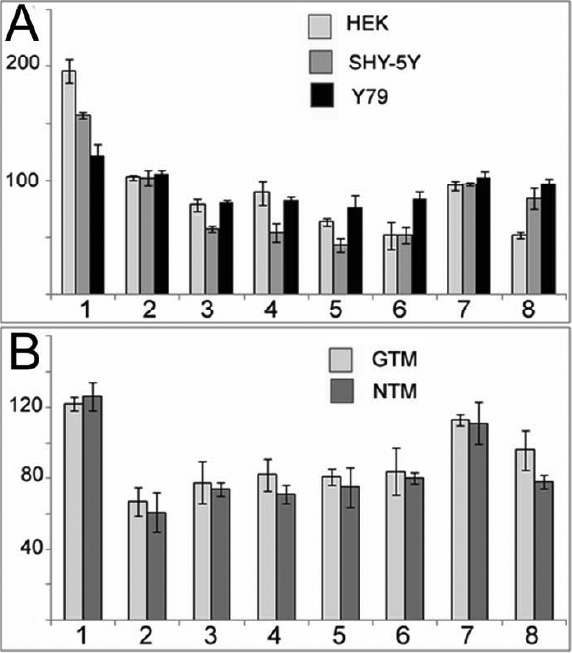
Effect of *CAV-1* promoter modification on the expression level in LUC system. Cells were transfected with 1 µg pGL3 promoterless LUC reporter plasmids inserted with intact or modified 2,465 bases *CAV-1* promoter region (pGL3-CAV-1 promoterA or pGL3-CAV-1promoterG). LUC activity was averaged. The SEM is indicated by the error bars. One – ratio of activity of construct with A to activity of construct with G in position −2,388 of *CAV-1* promoter; 2 – 8 putative cis-elements were deleted from *CAV-1* promoter and the activity of the construct with deleted sequence was expressed in percentage to the unmodified construct. The following putative TF-binding sites were deleted: 2– NFκB (position −1537 −1527); 3 - HNF-3/Fkh homolog (position - 889 −882); 4 - POU-IV position (−807 −800); 5 – Sp-1 (position −236 −226); 6 – Ets (−182–174); 7 - SP-1 (position −124–115); 8– NFkB (position −40 −28). Other details are presented in Table 2. **A**: The effect of CAV-1 promoter modification on LUC activity in HEK-293, SH-SY5Y and Y79 cells. **B**: The effect of *CAV-1* promoter modification on LUC activity in NTM-5 and GTM-3 cells.

Following transfection in GTM and NTM cells, the construct containing A in position −2,388 possessed stronger luciferase activity (122% and 125.8%) compared to the construct containing G (100%; Figure 6, bar 1).

Deletion of putative TF-binding sites 3–6 caused a ~10%–30% reduction of transcription activity in all cell types tested (Figure 6). For some of the tested TF-binding sites the effect was different in TM cells and other types of cells. For example, deletion of cis-element nuclear factor kappa-light-chain-enhancer of activated B cells (NFκB) (−1,537 −1,527, bars 2 in Figure 6) caused statistically significant reduction of expression (p<0.05) by ~25% in both types of TM cells (Figure 6B), but did not alter it in HEK, SHY-5Y and Y79 cells (Figure 6A). Deletion of Sp1 binding site (−236 – 226, bars 5) and E-twenty six (ETS) binding site (−182–174, bars 6 in Figure 6) caused only slight reduction of the promoter activity in both types of TM cells, but caused statistically significant reduction (p<0.01) in SHY-5Y cells (43.6 and 52% of residual activity, respectively). In contrast, the deletion of the cis-element 7 (Sp1 binding site −124–115) did not alter the level of transcription in HEK, SHY-5Y and Y79 cells, but slightly increased it in both types of TM cells (Figure 6B).

## Discussion

Our data show that CAV-2 expression varies between individuals, whereas the level of CAV-1 expression is similar (Figure 2). CAV-1 and CAV-2 have a similar perinuclear punctate appearance in NTM and GTM cells (Figure 2A-F) and are colocalized in dot-like structures (Figure 2D-F). The results of intracellular colocalization of CAV-1 and CAV-2 in TM cells are similar to the data obtained previously in adipose tissues and other tissue types [14,16]. As was suggested earlier, colocalization of CAV-1 and CAV-2 within a single cell point to a different role they might play in cellular physiology, for example in their effect on intracellular signaling through heterotrimeric G proteins [14].

We used two commonly known glaucoma promoting agents, i.e.TGF-β2 and DEX to evaluate their effect on caveolins.TGF-β2 is known to be strongly upregulated in aqueous humor of POAG patients [41], whereas DEX is a glucocorticoid hormone which influences many aspects of cell physiology. In addition, DEX is a widely used topical ocular anti-inflammatory drug which exerts its therapeutic effects by binding to the glucocorticoid receptor, leading to the modulation of gene expression by trans- repression (i.e., regulating transcriptional regulators such as nuclear factor NFκB [42]). However, it can also cause an increase in IOP and subsequently, glaucoma [43].

In experiments with immortalized normal and glaucomatous TM cells DEX caused considerably higher upregulation of CAV-1 expression in GTM-3 cells compared to normal NTM-5 cells where the effect was moderate (Figure 4A).

The level of CAV-1 phosphorylation was lower in glaucomatous TMs when compared to normal TM cells (Figure 5). This reduction in Tyr14 phosphorylation may have a significant impact on cell physiology, because phosphorylation of this tyrosine residue alters the properties and intracellular localization of CAV-1 [38,39]. For example, phosphorylation at Tyr14 is important for CAV-1 binding to c-Src/Grb7 signaling complex [44]. CAV-1 undergoes Tyr14 phosphorylation and then serves as a scaffolding protein to recruit SH2-domain containing proteins. This in turn may increase the anchorage independent growth of cells [44].

Overall, our results show a reducing trend in caveolin protein expression, including reduced phosphorylation, in cells and tissues derived from glaucoma patients. However there was considerable variation among the different samples and the cell lines used. This might be due to inherent genetic diversity of the donor individuals as well as the fact that transformed cell lines often do not mimic the original state during in vitro experiments. Further studies are needed to show whether this trend is linked to the pathogenicity of POAG.

Interestingly, CAV-1 scaffolding domain (amino acids 81–100) is able to bind α-synuclein – a member of the synuclein family implicated in the process of neuro-degeneration. Such physical interaction between CAV-1 and α-synuclein may regulate α-synuclein-mediated actions on cell death, processes known to be involved in synucleinopathies [46-48]. However, CAV-1 cannot be considered as a protein indispensable for the internalization of α-synuclein [49]. As has been shown earlier, both α- and γ-synuclein are expressed in TM cells and participate in glaucomatous alterations [31,32,45]. Further studies are required to reveal whether CAV-1 – synuclein interactions play a role in glaucomatous interactions in TM cells. Another interesting aspect of the interaction between members of the synuclein family and CAV-1 is that α- synuclein upregulates expression of CAV-1 which might be important in the pathogenesis of neurodegenerative disorders [46-48].

Transcriptional regulation plays an important role in determining the level of caveolin expression in normal and disease states [21-24,49-51]. A bioinformatics search using the software PROMO has shown, that the common sequence variant (SNP) near *CAV-1* [5] might create additional TF binding sites in the upstream region of *CAV-1*. In the presence of A at position −2,388 a homeobox is created with a sequence of GTATT GTATT which may serve as a binding site for new TFs [52-54]. The binding of HoxD9, HoxD10, and HNF-3A has been predicted by analysis of this homeobox using the software PROMO search. Binding of TFs to this homeobox in turn might increase the level of gene expression detected in LUC system (Figure 6). As shown previously, elevated levels of CAV-1 expression may cause organ-specific abnormalities [55] and exert a negative effect on cell physiology, for example by increasing sensitivity to environmental stress [56].

Surprisingly, no significant variability in CAV-1 levels in TM cells taken from five POAG patients and four controls was found (Figure 2A). On the contrary, CAV-2 levels in these cells were more variable. Since information about the polymorphisms in the area of caveolins genes for the individuals whose TM cells were analyzed was unavailable, it was difficult to explain why no correlation was found between polymorphic variation in the *CAV-1* promoter (Figure 6) and the level of variability in the amount of CAV-1 in TM cells from patients (Figure 2A). One of the possible explanations might be that all five patients had one allele, most probably, a major allele. Alternatively, although the polymorphic marker is located at a much longer distance from *CAV-2* than from *CAV-1* and it is in the 3′-end of *CAV-2*, one cannot exclude the idea that this marker may affect the expression level of CAV-2.

Another interesting finding concerning CAV-2 in addition to variable level of its expression in samples from different patients (Figure 2B) is the presence of high molecular weight aggregates which were previously characterized as heterooligomers containing CAV-1 and CAV-2 [35].

Elevated levels of CAV-1 and CAV-2 have been associated with several forms of cancer, Alzheimer disease, and other human diseases [57,58]. One of the mechanisms underlying their involvement in pathology is linked to alterations of the cholesterol distribution in the cellular plasma membrane leading to the dysregulation of cholesterol homeostasis. A dysregulated expression of caveolin may also be responsible for alterations in caveolin-dependent signal transduction in cells [59].

Analysis of the effect of deletions and substitutions in the upstream region of *CAV-1* on LUC expression demonstrated that the substitution of G with A (SNP described by Thorleifsson and coauthors [5]) as well as deletions of several putative TF-binding sites affected gene expression. The presence of A in position −2,388 ensured a higher level of promoter activity than G in all cell types tested (Figure 6). The deletion of putative TF-binding sites affected transcriptional activity in a cell-type specific manner.

Deletion of putative TF-binding sites 3–6 for HNF-3, Pou-IV, Sp1 (−236 −226), and Ets caused a ~10%–30% reduction of transcription activity in all cell types tested (Figure 6, bars 3–6). Deletion of cis-element 7 (Sp1 −124–115) did not alter the level of transcription in HEK, SHSY-5Y, and Y79 cells and slightly increased it in both types of TM cells (Figure 6B). In NTM cells the effect of deletion of TF-binding sites was more pronounced than in GTM cells.

In summary, the data presented here demonstrate new mechanisms of *CAV-1* and *CAV-2* regulation which may play an important role in glaucomatous alterations in TM cells. A recently described A/G polymorphism in the *CAV-1* promoter [5] was found to affect the level of expression directed by the *CAV-1* upstream region in LUC system. Another interesting finding is a significant level of variability of *CAV-2* expression in TM cells from different individuals, compared to a similar level of *CAV-1* expression. Further studies are required to reveal whether the variability in *CAV-2* expression is relevant to glaucoma pathogenesis.
